# Specialty experience in geriatric medicine is associated with a small increase in knowledge of delirium

**DOI:** 10.1093/ageing/aft159

**Published:** 2013-10-16

**Authors:** Rodric Peter Llewelyn Jenkin, Patrick Musonda, Alasdair M. J. MacLullich, Phyo Kyaw Myint, Daniel H. J. Davis

**Affiliations:** 1 Department of Medicine for the Elderly, St Mary's Hospital, Praed Street, London W2 1NY, UK; 2 Norwich Medical School, University of East Anglia, Centre for Infectious Disease Research, NorwichUK; 3 Edinburgh Delirium Research Group, University of Edinburgh, Edinburgh, UK; 4 Academic Department of Medicine for the Elderly, University of East Anglia, Norwich, Norfolk, UK; 5 Department of Public Health and Primary Care, University of Cambridge, Cambridge, UK

**Keywords:** delirium, survey, geriatric, doctor, specialty, older people

## Abstract

**Background:** delirium is underdiagnosed and undertreated. Understanding of delirium among doctors in medical and ICU settings has previously been shown to be low. We hypothesised that junior doctors who had gained experience in geriatrics, neurology or psychiatry may have an increased knowledge of delirium.

**Methods:** we used data from a large multi-centre study of junior doctors conducted between December 2006 and January 2007 which is, to date, the largest survey of understanding of delirium among junior doctors. The original survey used a questionnaire within which certain key items led to a correct or incorrect answer. Total correct answers were recorded giving a maximum total knowledge score of 17 for each participant. The relationship between total knowledge score achieved on the questionnaire and time since qualification; specialty experience in geriatric medicine, psychiatry and/or neurology and self-reported experience with the Confusion Assessment Method (independent variables) were modelled using linear regression.

**Results:** around half (53.2%; 399 of 750) of those surveyed stated that they had experience in geriatric medicine. In contrast only 4.1 and 8.0% of respondents had experience in psychiatry and neurology, respectively. Experience in geriatric medicine was significantly associated with a modest increase in correct answers (4.7 versus 4.3 points, *P* = 0.020). No other variables were significantly associated with better scores.

**Conclusion:** experience in geriatric medicine leads to a small improvement in understanding of delirium among junior doctors.

## Introduction

Delirium is an acute neuropsychiatric syndrome, occurring in 11–42% of elderly in-patients [1]. The presence of delirium is associated with prolonged hospital stay, higher rates of institutionalisation and increased mortality [2]. Furthermore, delirium is associated with accelerated cognitive decline in those with pre-existing dementia and a significantly increased risk for the development of dementia in those with normal cognition [3, 4]. Despite its high prevalence and major clinical significance, delirium is consistently under-recognised [1, 5].

Previous studies have examined doctors' understanding of delirium in the medical inpatient and ICU settings [6–8]. These studies have shown that the understanding of delirium appears to be poor in comparison with other common medical conditions such as acute coronary syndrome or pneumonia. In addition, there is a consistent discrepancy between participants' perception of the importance of delirium and their understanding of delirium; while the respondents acknowledged delirium is highly prevalent and important, they lacked understanding and were unfamiliar with delirium assessment tools.

Whether the knowledge of delirium among junior doctors is directly related to duration of their medical training or specialties in which they have worked has not previously been examined. We hypothesised that attachments in geriatrics, neurology or psychiatry and total time working as a doctor may be associated with a greater knowledge and understanding of delirium.

## Subjects and methods

We used data from a multicentre survey conducted in the UK between 1 December 2006 and 31 January 2007 which is, to date, the largest survey of doctors' understanding of delirium. The methods of the survey have been previously reported [7]. In brief, a questionnaire was designed to test knowledge of delirium prevalence, diagnostic criteria using DSM-IV, use of specific screening tools, association with adverse outcomes and pharmacological management. A convenience sample of junior doctors working in acute medical specialties and emergency medicine was taken at each participating trust and responses from 784 doctors were received in total. The 34 Acute trusts involved were recruited through contacts in the British Geriatrics Society. Participants filled in the questionnaire at the point of approach and no site investigator reported any refusals.

In addition to answering the questionnaire, participants were asked to report whether they had postgraduate specialty experience in geriatric medicine, psychiatry and/or neurology and the number of months since they originally qualified as a doctor.

### Statistical analyses

Within the questionnaire, certain key items led to a correct or incorrect answer, for each either a score of 1 (for a correct answer) or a score of 0 (for an incorrect answer) was given from which a total score out of 17 was derived. Typical knowledge questions included: ‘Using DSM-IV, which of the following features are essential to a diagnosis of delirium?’; ‘Patients with delirium most commonly display reduced motor activity and lethargy (true/false?)’. ‘Assuming no-contraindications, what would you consider to be an appropriate starting dose of haloperidol in a 70-year-old with severe agitation in whom behavioural management has been unsuccessful?’ Answers were given equal weighting in the score; missing answers were regarded as incorrect. The unadjusted relationships between specialty exposure and median knowledge scores were assessed using the Mann–Whitney U test. The relationship between total knowledge score (dependent variable) and time since qualification; specialty experience in geriatric medicine, psychiatry and/or neurology and self-reported experience with the Confusion Assessment Method (CAM) (independent variables) were modelled using linear regression, with random-effects allowed for recruitment centre to account for the clustered nature of the data. All analyses were conducted in Stata 12.1 (StataCorp, USA).

## Results

Around half (53.2%; 399 of 750) of those surveyed stated that they had experience in geriatric medicine. In contrast only 4.1 and 8.0% of respondents had experience in psychiatry and neurology, respectively. Use of the CAM was reported by 8.6%.

**Figure 1.**
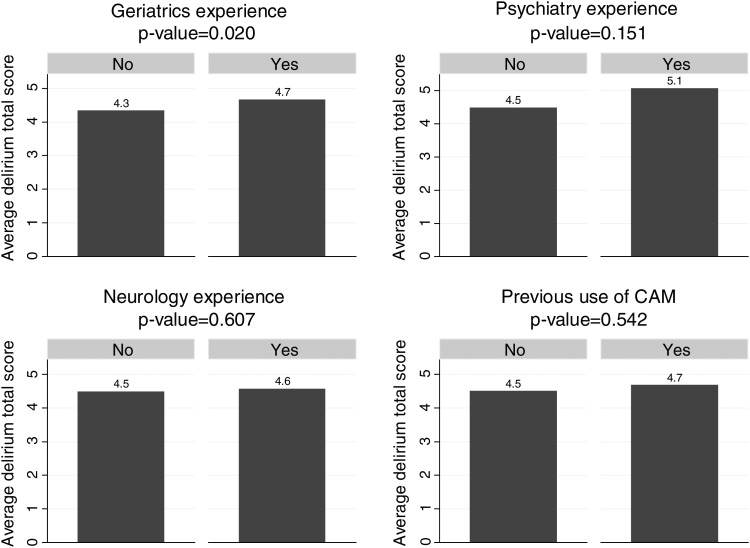
Average delirium scores by specialty experience. Bar charts showing differences in knowledge score, by specialty exposure and previous use of the Confusion Assessment Method.

Scores on the knowledge of delirium items followed a normal distribution. The median score was 4 out of a maximum possible score of 17. Figure 1 shows the unadjusted relationships between specialty experience and knowledge score. Experience in geriatric medicine was significantly associated with a modest increase in correct answers (4.7 versus 4.3 points, *P* = 0.020). No other variables were significantly associated with better scores.

The linear regression models showed that self-reported experience in geriatric medicine was significantly associated with an extra 0.29 correct answers after adjusting for centre when compared with those without specialty experience (*P* = 0.029) (Table 1). In addition, months since qualification and experience in psychiatry were associated with slightly better scores before adjusting for centre. However, after accounting for clustering in the data, these variables were no longer significant. Post-estimation of testing of model residuals showed no violation of the model assumptions, with non-constant residual variance (*P* = 0.21).

**Table 1.** Summary values of key characteristics and estimates from linear mixed models, with and without adjustment for survey centre

Explanatory variable	Answering ‘Yes’, *n* (%)	Unadjusted, slope (95% CI)	*P*-value	Adjusted, slope (95% CI)	*P*-value
No. of months experience	N/A	0.006 (0.002, 0.010)	0.001	0.004 (−0.000, 0.008)	0.063
Experience in geriatrics	399/750 (53.2%)	0.306 (0.073, 0.539)	0.010	0.291 (0.030, 0.553)	0.029
Experience in psychiatry	29/711 (4.1%)	0.600 (−0.000, 1.194)	0.050	0.479 (−0.187, 1.145)	0.159
Experience in neurology	57/712 (8.0%)	0.066 (−0.369, 0.501)	0.766	−0.157 (−0.672, 0.358)	0.549
Previous use of CAM	64/748 (8.6%)	0.176 (−0.241, 0.593)	0.408	0.015 (−0.456, 0.487)	0.949

CAM, Confusion Assessment Method. The adjusted slopes account for clustering by centre. The slopes show the coefficients from the model output and can be interpreted as the additional number of correct answers given self-reported clinical experience: per extra month of experience since qualification; for previous experience in (geriatric medicine/psychiatry/neurology) compared with no experience in (geriatric medicine/psychiatry/neurology) and for person with previous experience of the Confusion Assessment Method (CAM) compared with those without previous experience of the CAM.

## Discussion

We confirm that the overall knowledge of delirium appeared low in junior doctors at the time the survey was conducted. Experience in geriatric medicine was associated with a higher level of knowledge; however, the difference was small. Experience in neurology, psychiatry, use of the CAM and total months since qualification were not significantly associated with an improved knowledge of delirium. The small proportion with experience in neurology and psychiatry may have resulted in lack of power to detect any differences in knowledge.

This is the first study to examine an association between experience in geriatric medicine and knowledge of delirium. The study benefits from its large sample size and that the participants were drawn from trusts across the UK.

There are a number of limitations. It was a convenience sample so there is a possibility of selection bias, although this may be mitigated to an extent by the large sample size. Although ‘experience in geriatrics’ referred to experience since qualification rather than exposure as a student, we did not ask participants to clarify what their specific experience had entailed. It is possible that a subset of doctors who have worked in posts with an emphasis on acute geriatric care or worked in specific acute geriatric units may have had a greater increase in level of knowledge of delirium and our analysis would not have detected this. We also did not ask how long ago they worked in geriatric medicine or whether they are currently working in geriatric medicine. As such, it is possible that a placement in geriatric medicine provides a more significant increase in knowledge of delirium, but that this is not sustained.

It is possible that insufficient emphasis was placed on the diagnosis of delirium in medical practice in general at the time of our survey. In the UK, the 2009 curriculum for Core Medical Trainees (including revisions up to August 2012) required knowledge coming under the heading of ‘Acute confusion/Delirium’. Although it specifically states that trainees should know the predisposing and precipitating factors for delirium, and the initial and subsequent investigations required, no mention is made of making the diagnosis of delirium or knowing the diagnostic criteria. This is in contrast to the preceding section in the same document where for chest pain, an appropriate emphasis on the diagnostic process is included (http://www.jrcptb.org.uk/trainingandcert/Documents/2009%20CMT%20framework%20(revised%20Aug%202012).pdf).

The curriculum for more senior trainees specialising in geriatric medicine has ‘diagnostic criteria for delirium’ as part of the expected knowledge to be acquired (http://www.gmc-uk.org/geriatric_curriculum_2010.pdf_32486221.pdf). Although it is questionable how much the specialty curricula directly influence junior doctors' learning, it would seem appropriate to include the diagnostic criteria for delirium as a knowledge requirement for CMT doctors in the next iteration of the curriculum. This would parallel the expectation that a core medical trainee should know how to diagnose a heart attack, stroke or pneumonia.

The publication of the 2010 NICE guidelines for delirium may have provided a stimulus to greater understanding of delirium among trainees, giving a clear national reference for the identification, investigation and management of patients with delirium (http://guidance.nice.org.uk/CG103/NICEGuidance/pdf/English). A further significant policy shift initiative which may lead to an improvement in the recognition of delirium is the implementation of the dementia CQUIN (Commissioning for Quality and Innovation) target. This requires trusts to perform a cognitive assessment on all patients admitted as an emergency who are over the age of 75 with an aim to increase identification of patients with delirium and dementia. As the summary guidance for the dementia CQUIN points out, ‘[identifying] all patients with cognitive impairment will improve the detection of delirium’ (https://www.wp.dh.gov.uk/cno/files/2012/05/CQUINN-safety-thermometer-guidance.pdf).

In summary, specialty experience in geriatric medicine provides a small improvement in knowledge of delirium, and increased emphasis on training in this area may lead to this effect being strengthened. Repeating the original survey in light of the NICE guidelines and increasing profile of delirium would be of great interest.

**Key points**

- Delirium is underdiagnosed and undertreated.
- Understanding of delirium among junior doctors is low.
- Experience of Geriatric Medicine leads to a modest increase in understanding of delirium.

## Conflicts of interest

None declared.

## Funding

D.D. is funded by the Wellcome Trust as a Research Training Fellow. R.J., P.M., A.M. and P.K.M. have no funding acknowledgments to declare.
